# DIAMOND2GO: rapid Gene Ontology assignment and enrichment detection for functional genomics

**DOI:** 10.3389/fbinf.2025.1634042

**Published:** 2025-08-15

**Authors:** Christopher Golden, David J. Studholme, Rhys A. Farrer

**Affiliations:** ^1^ Medical Research Council Centre for Medical Mycology at the University of Exeter, Department of Biosciences, Faculty of Health and Life Sciences, Exeter, United Kingdom; ^2^ Biosciences, University of Exeter, Exeter, United Kingdom

**Keywords:** functional genomics, software, diamond, Gene Ontology, enrichment analyses

## Abstract

DIAMOND2GO (D2GO) is a high-speed toolset for assigning Gene Ontology (GO) terms to genes or proteins based on sequence similarity. Leveraging the ultra-fast alignment capabilities of DIAMOND, which is 100 to 20,000 times faster than BLAST, D2GO enables rapid functional annotation of large-scale datasets. D2GO maps GO terms from pre-annotated sequences in the NCBI non-redundant database to query sequences. During benchmarking, D2GO assigned over 2 million GO terms to 98% of 130,184 predicted human protein isoforms in under 13 min on a standard laptop. In addition to annotation, D2GO includes an enrichment analysis tool that allows users to identify significantly overrepresented GO terms between subsets of sequences. We compared D2GO against two widely used tools, Blast2GO and eggNOG-mapper, and observed substantial differences in the number and type of annotations produced. These discrepancies reflect varying sensitivities and specificities across tools and suggest that using multiple methods in tandem may improve overall annotation coverage. D2GO is open-source and freely available under the MIT license at https://github.com/rhysf/DIAMOND2GO.

## Introduction

The Gene Ontology (GO) provides a structured, controlled vocabulary for describing gene product functions across species (Ashburner et al., 2000). GO is organized into three primary categories: molecular function (MF), which describes the specific biochemical activities of gene products; cellular component (CC), which indicates where these activities occur in the cell; and biological process (BP), which captures broader physiological events involving multiple molecular activities (Ashburner et al., 2000). GO terms are arranged in a loosely hierarchical structure, where more specific “child” terms are linked to broader “parent” terms. For example, the MF for GO:0004375 glycine dehydrogenase (decarboxylating) activity is a more-specific child of GO:0003824 catalytic activity. A single child term may belong to several parent terms, reflecting the complex and interconnected nature of biological functions.

GO is developed and maintained by the GO Consortium (Ashburner et al., 2000), which curates the GO knowledgebase (Aleksander et al., 2023) as part of a larger initiative by the Open Biological and Biomedical Ontologies (OBO) Foundry (Smith et al., 2007). The OBO Foundry oversees a wide range of ontologies, such as the Cell Ontology, the Foundational Model of Anatomy, and the Plant Ontology (Ashburner et al., 2000). Although the GO is a widely used framework for functional annotation, other resources such as Pfam (Protein Families) (Finn et al., 2014) and the Kyoto Encyclopedia of Genes and Genomes (KEGG) (Kanehisa and Goto, 2000) also provide functional insights. Functional annotations are often inferred for newly predicted genes or proteins through sequence similarity, comparative genomics, or structural features.

Several tools have been developed to assign GO terms to protein or nucleotide sequences, with Blast2GO (B2GO) being one of the most widely used. B2GO applies sequence similarity searches using either BLAST or the faster DIAMOND algorithm (Buchfink et al., 2015) to identify homology between input sequences and experimentally annotated proteins (Conesa et al., 2005). B2GO supports searches against custom user-defined databases or established reference datasets such as the NCBI non-redundant (nr) database or UniProtKB/Swiss-Prot (Bairoch and Apweiler, 2000). These searches can be run locally or through remote services like CloudBLAST (Matsunaga et al., 2008), the NCBI QBLAST server, or Amazon Web Services (AWS) BLAST. GO term assignment in B2GO is based on a multi-step algorithm that integrates sequence similarity results with InterProScan (Hunter et al., 2009) domain predictions. The assignment process considers factors such as alignment quality, coverage of the query-hit match, the source and curation level of the database, the structure of the GO hierarchy, and the annotation evidence of the matched sequences.

In addition to the GO-term assignment, B2GO offers a suite of features for visualization and functional analysis. B2GO is integrated into the broader OmicsBox platform (Bioinformatics Software OmicsBox, 2024), which provides a user-friendly graphical user interface for genome and transcriptome analysis—particularly appealing to researchers without formal bioinformatics training. By default, B2GO performs similarity searches using online BLAST services, typically against the nr protein database. Although this approach provides access to a broad and curated reference set, it is often slow, with searches taking several minutes per sequence or batch, posing a significant bottleneck for large datasets. Although users can install a local database to improve performance, this option requires substantial storage, setup time, and technical expertise. For large-scale queries, such as complete proteomes from newly sequenced genomes, this limitation can significantly delay downstream analyses. Another important consideration is that B2GO/OmicsBox is no longer freely available. Although a 7-day free trial is offered, continued use requires a paid license, even for academic users.

Beyond B2GO, a range of other tools and approaches have been developed for the GO-term assignment. Some leverage machine learning models trained on diverse features such as predicted protein domains, GO-term co-occurrence patterns, and phylogenetic profiles (You et al., 2018; Fa et al., 2018). These methods aim to improve functional prediction accuracy by incorporating biological context beyond direct sequence similarity. eggNOG-mapper (Huerta-Cepas et al., 2017) is a widely used tool that assigns GO terms based on precomputed orthology relationships from the EggNOG database. By mapping query sequences to orthologous groups, it infers function through high-confidence evolutionary relationships. In contrast, Wei2GO (Reijnders, 2022) combines DIAMOND and HMMScan searches against the UniProtKB and Pfam databases to assign GO terms based on both sequence similarity and conserved domain architecture. Both eggNOG-Mapper and Wei2GO are freely available and open-access, making them accessible options for a wide range of genome annotation projects.

The Critical Assessment of Functional Annotation (CAFA) is a community-driven challenge designed to evaluate computational methods for protein function prediction. CAFA uses a time-delayed evaluation framework, in which predictions are submitted prior to the release of new experimental GO annotations, which are then used to benchmark a ground-truth set (Zhou et al., 2019). This approach enables objective and standardized comparison of annotation tools. In the most recent CAFA3 assessment, modest improvements in prediction performance were observed between 2016 and 2019 for MF and BP categories, but not for CC. The top-performing method in CAFA3 was GOLabeler, a machine learning-based approach that integrates features such as GO term frequency, sequence alignment, and amino acid trigrams (You et al., 2018). However, GOLabeler is currently not publicly available, and its official website has been offline since at least August 2024.

Once GO terms are assigned, they can be leveraged in a variety of downstream analyses using a wide range of dedicated tools (Shahzad et al., 2015). For example, PANTHER facilitates evolutionary and functional classification of protein-coding genes, allowing researchers to explore gene families, pathways, and biological processes across species (Thomas et al., 2022). The AmiGO web portal enables users to search, filter, visualize, and analyze GO annotations within the official GO database (Carbon et al., 2009). For more advanced modeling, GO-Causal Activity Modeling (GO-CAM) links individual GO terms into structured, interpretable networks that represent biological pathways and regulatory relationships (Thomas et al., 2019).

The release of DIAMOND in 2014 introduced a major leap in sequence alignment speed, achieving protein and translated DNA alignments 100 to 10,000 times faster than BLAST (Buchfink et al., 2015). This performance improvement offers a substantial advantage for time-consuming GO annotation workflows such as those used by B2GO. Additional gains in efficiency can be achieved by restricting searches to only those database sequences with existing GO annotations, reducing the search space to under 1 gigabase, and avoiding unnecessary alignments. By integrating the speed of DIAMOND with a lightweight, purpose-built annotation pipeline, we developed DIAMOND2GO (D2GO). D2GO can assign millions of GO terms to hundreds of thousands of proteins within minutes on a standard laptop (see Implementation section), offering an accessible and scalable solution for genome-wide functional annotation.

## Implementation

All analyses in this study were conducted on a 2021 MacBook Pro equipped with an Apple M1 Max CPU and 64 GB RAM. The NCBI non-redundant database was downloaded on 14 May 2023 and pre-processed to remove non-printable ASCII characters. Associated gene and GO-term mappings were obtained from the NCBI gene2accession and gene2go files on 20 July 2023 (https://ftp.ncbi.nih.gov/gene/DATA/).

**FIGURE 1:**
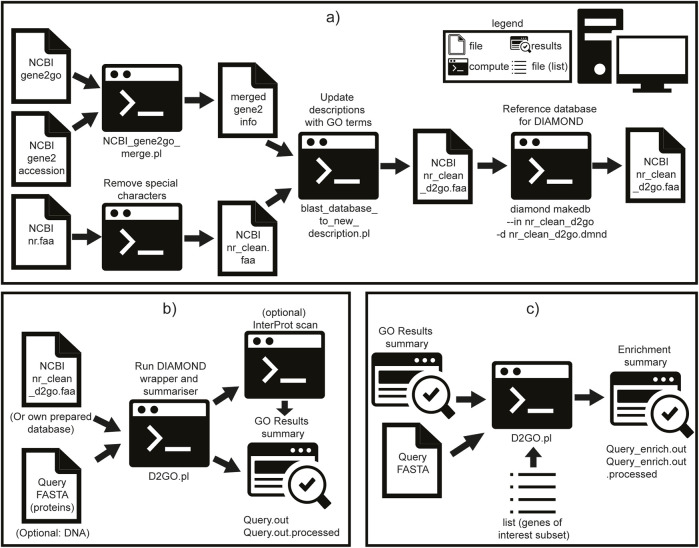
Schematic overview of the steps to construct a new database for DIAMOND2GO (D2GO), run D2GO, and perform enrichment analysis. Symbols are described in the embedded legend. All scripts and steps are included as part of software. **(a)** Pre-prepared D2GO database, **(b)** run D2GO, and **(c)** run D2GO enrichment.

GO terms were merged with gene accessions using the D2GO utility script ncbi_gene2go_merge.pl and added to sequence descriptions via blast_database_to_new_description.pl. The resulting annotated database was indexed using DIAMOND makedb (Buchfink et al., 2015). This pre-processed and indexed database is included in the DIAMOND2GO GitHub repository as a Git Large File Storage (LFS) file; Git LFS must be installed as a prerequisite. Instructions for rebuilding this database are provided in Figure 1a. A Docker container is provided, with all required dependencies pre-installed, and is configured to run D2GO without additional setup. Instructions for using the container are included in the GitHub README. D2GO is freely available under the MIT license from https://github.com/rhysf/DIAMOND2GO.

D2GO acts as a lightweight wrapper around DIAMOND, adding mapping and output-parsing functionality. It currently supports both protein (BLASTP) and translated nucleotide (BLASTX) searches. The default settings include sensitivity set to ultra-sensitive, E-value cutoff: 10^−10^, and max target sequences: 1. A schematic of the D2GO pipeline is shown in Figure 1b.

The D2GO functional annotation pipeline consists of four main steps: 1. DIAMOND alignment is performed using user-defined parameters (or defaults listed above). 2. Result summarization generates a tab-delimited file containing the gene name, species, associated GO terms, and evidence codes. GO terms tagged with NOT (e.g., NOT located_in) are excluded for clarity. Redundant GO terms per gene are collapsed by retaining only the match with the lowest E-value. 3. (Optional) InterProScan preparation: queries are filtered (optionally selecting only DIAMOND non-hits), STOP codons are removed, and sequences are batched into groups of 500. 4. (Optional) InterProScan annotation is run using the bundled iprscan5.pl script. The results are parsed and merged with the previous D2GO output to produce a combined annotation.

D2GO was tested on two datasets: 1. All predicted human proteins and splice variants from NCBI GenBank (GRCh38.p14, assembly: GCA_000001405.29) (Lander et al., 2001; International Human Genome Sequencing Consortium, 2004). 2. All predicted proteins of the chytrid fungus *Batrachochytrium salamandrivorans* (*Bsal*), downloaded from GenBank (assembly: GCA_002006685.2) (Wacker et al., 2023). The search parameters used were as follows: sensitivity set to ultra-sensitive, max target sequences: 1, and E-value cutoff: 1e-5. For comparative benchmarking, B2GO v6.0.3 was run on the *Bsal* dataset as previously described (Wacker et al., 2023). eggNOG-mapper v2.1.12–3 was used with the following parameter values: m diamond--decorate_gff--excel--cpu 12 --override. InterProScan was integrated using the bundled iprscan5.pl script. A Venn diagram showing the overlap of results among tools was generated using InteractiVenn (Heberle et al., 2015). For GO-term enrichment analysis, significance was determined using a two-tailed Fisher’s exact test followed by Storey–Tibshirani FDR correction (q-value <0.05) (Storey and Tibshirani, 2003).

## Results

We present DIAMOND2GO (D2GO), a fast and open-access tool for assigning GO terms without subscription or license costs. To assess its performance, we annotated all 130,184 predicted human proteins, including splice variants. D2GO assigned 2,060,956 GO terms to 127,625 proteins (>98%) in just 12 min and 35 s on a laptop (see Implementation). In comparison, Blast2GO (B2GO) required several days on the same dataset, while EggNOG-mapper completed the task in 29 min and 38 s.

We evaluated D2GO alongside B2GO and eggNOG-mapper using the *Bsal* proteome (Wacker et al., 2023), running D2GO with eight different parameter sets (see Supplementary Table S1). Using the compare_go_tools.pl script (bundled with D2GO), we assessed both the number of genes with GO terms and the total number of GO terms assigned. D2GO assigned between 68,236 and 203,241 GO terms to between 5,756 and 7,729 genes (53%–71% of all 10,867 genes), depending on the parameters used. eggNOG-mapper assigned 467,171 GO terms to 4,934 genes (45%). B2GO assigned 34,408 GO terms to 8,683 genes (80%). Interestingly, eggNOG-mapper assigned the highest number of unique GO terms (88%–94% not found in other tools), followed by D2GO (45%–69%) and B2GO (28%–36%).

**FIGURE 2:**
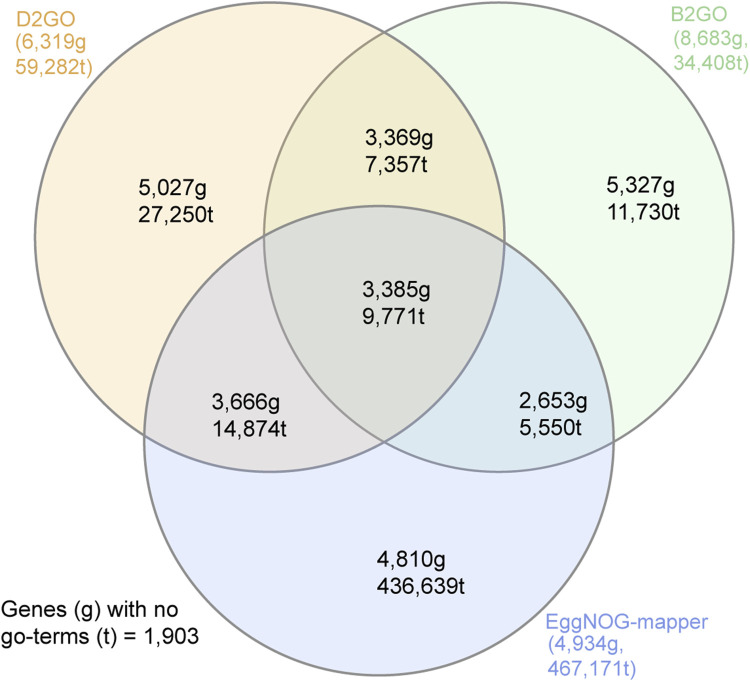
Venn diagram showing the number of *Bsal* genes (g) that were assigned GO terms by D2GO (recommended parameters), B2GO, and eggNOG-mapper. The total number of GO terms (t) that were assigned to *Bsal* genes by each tool is also shown.

D2GO’s annotation output varied depending on sensitivity and alignment parameters. Using e-value < 1e-5 and max target = 1, GO terms were assigned to 57% of genes. Switching to ultra-sensitive alignment increased coverage to 64% but increased runtime from <10 s to approximately 9.5 min. Based on this trade-off, we set ultra-sensitive, e-value = 1e-5, and max target = 1 as the default parameters, balancing speed and coverage. Gene and GO term overlaps with B2GO and eggNOG-mapper, using these defaults, are shown in Figure 2.

D2GO includes a wrapper for InterProScan, allowing extended annotation of sequences that lack DIAMOND hits. InterProScan added GO terms to an additional 7% of genes (increasing the total to 71%). However, the runtime increased substantially: ∼3 h for only DIAMOND non-hit genes and ∼17 h when run on all genes. Minimal additional GO term overlap was observed between D2GO and B2GO (Supplementary Table S1). Thus, although InterProScan expands coverage, it significantly reduces D2GO’s speed advantage. We recommend using it only when completeness is prioritized over runtime.

To evaluate annotation quality, we examined results for a well-characterized gene: DNA polymerase alpha subunit pol12 (BSLG_001742). Both D2GO and B2GO identified the same three core GO terms. D2GO with InterProScan identified two additional parent terms (1 BP and 1 CC) (Supplementary Table S2). Even though B2GO matched a different *Bsal* gene (BSLG_01791) and D2GO was assigned to *Xenopus laevis* (due to *Bsal* absence in gene2go), the assigned GO terms were identical, highlighting D2GO’s effectiveness despite differences in database content.

To test downstream usability, we used the D2GO utility test_enrichment.pl on all human genes with the term “polymerase” in their FASTA description (see Figure 1C). The top 10 enriched GO terms were all polymerase-related (Supplementary Table S3), confirming the tool’s ability to detect biologically relevant signals and suggesting utility for less-characterized gene groups.

## Discussion

D2GO offers a fast and accessible alternative to existing functional annotation tools such as B2GO and eggNOG-mapper. In our benchmark, D2GO demonstrated significantly improved speed over both tools while also being freely available, like eggNOG-mapper. This makes it particularly suitable for large-scale projects or rapid exploratory analysis where annotation time can be a limiting factor.

Without a gold standard for GO annotations, it remains difficult to determine absolute accuracy when discrepancies arise among tools. However, the variability we observed in GO-term assignment across D2GO, B2GO, and eggNOG-mapper underscores that each tool carries unique sensitivity and specificity profiles. For instance, we noted that D2GO’s results were sensitive to user-defined parameters: increasing the number of accepted hits increased GO-term coverage but also yielded a higher number of uniquely assigned terms, potentially reflecting lower specificity. In contrast, restricting to top-hit matches or applying more stringent E-value thresholds led to fewer assignments and better consistency with other tools, indicating a trade-off between coverage and stringency. D2GO will also benefit from independent evaluation in future community-driven assessments, such as CAFA, to establish its predictive value under standardized benchmarks.

Our results suggest that consensus-driven annotation, where results from multiple tools are combined, may improve confidence in GO-term assignments, an approach that is increasingly advocated (Gaudet et al., 2011; Salzberg, 2019). In this context, D2GO could be integrated as a complementary tool within existing annotation pipelines. For example, workflows incorporating multiple annotation sources—either in a voting system or tiered schema—could leverage D2GO’s speed for initial or broad annotation sweeps, followed by more conservative refinement with tools like B2GO or domain-specific curation. By enabling faster and higher-throughput GO annotations, D2GO can accelerate downstream applications such as functional enrichment analysis, pathway discovery, and systems biology investigations. These annotations play a critical role in diverse fields, including disease genomics and biomarker discovery (Škorjanc et al., 2025; Köhler et al., 2009).

D2GO’s support scripts for enrichment analysis streamline integration into multi-omics workflows, making it a practical tool not only for standalone use but also for complementing more complex pipelines. With growing reliance on automated annotation in metagenomics, environmental sequencing, and transcriptomic analysis, D2GO’s balance of speed, flexibility, and usability positions it as a valuable addition to the functional annotation toolkit.

In conclusion, D2GO expands the landscape of GO annotation tools by offering a fast, open-source alternative with customizable parameters and enrichment-ready output. Its utility lies not only in its speed but also in its potential to complement existing tools, inform consensus-based annotations, and support a wide range of biological and biomedical research workflows.

## Data Availability

Publicly available datasets were analyzed in this study. These data can be found at https://github.com/rhysf/Diamond2GO.

## Supplementary material

SUPPPLEMENTARY TABLE S1 GO-terms assigned to Bsal proteins by D2GO using different settings, with or without InterPro (N=no, no_hits=run on genes with no DIAMOND hits, all=run on all genes). Overlap with B2GO is presented for comparison.

SUPPPLEMENTARY TABLE S2 GO-terms identified for the DNA-directed DNA polymerase alpha subunit pol12 (locus ID BSLG_001742) in Bsal by B2GO and D2GO (using InterProScan on all genes). GO-term sub-ontologies are Biological Process (BP), Cellular Component (CC) and Molecular Function (MF). Parental terms of other GO terms identified for this gene are shown.

SUPPPLEMENTARY TABLE S3 Top 10 significant GO terms for human genes that include the word ‘polymerase’ in the description.
